# A scoping study to identify opportunities to advance the ethical implementation and scale-up of HIV treatment as prevention: priorities for empirical research

**DOI:** 10.1186/1472-6939-15-54

**Published:** 2014-07-03

**Authors:** Rod Knight, Will Small, Basia Pakula, Kimberly Thomson, Jean Shoveller

**Affiliations:** 1 Interdisciplinary Studies Graduate Program, University of British Columbia, Vancouver, Canada; 2 Faculty of Health Sciences, Simon Fraser University, Burnaby, Canada; 3 School of Population and Public Health, University of British Columbia, Vancouver, Canada

## Abstract

**Background:**

Despite the evidence showing the promise of HIV treatment as prevention (TasP) in reducing HIV incidence, a variety of ethical questions surrounding the implementation and “scaling up” of TasP have been articulated by a variety of stakeholders including scientists, community activists and government officials. Given the high profile and potential promise of TasP in combatting the global HIV epidemic, an explicit and transparent research priority-setting process is critical to inform ongoing ethical discussions pertaining to TasP.

**Methods:**

We drew on the Arksey and O’Malley framework for conducting scoping review studies as well as systematic approaches to identifying empirical and theoretical gaps within ethical discussions pertaining to population-level intervention implementation and scale up. We searched the health science database PubMed to identify relevant peer-reviewed articles on ethical and implementation issues pertaining to TasP. We included English language articles that were published after 2009 (i.e., after the emergence of causal evidence within this field) by using search terms related to TasP. Given the tendency for much of the criticism and support of TasP to occur outside the peer-reviewed literature, we also included grey literature in order to provide a more exhaustive representation of how the ethical discussions pertaining to TasP have and are currently taking place. To identify the grey literature, we systematically searched a set of search engines, databases, and related webpages for keywords pertaining to TasP.

**Results:**

Three dominant themes emerged in our analysis with respect to the ethical questions pertaining to TasP implementation and scale-up: (a) balancing individual- and population-level interests; (b) power relations within clinical practice and competing resource demands within health care systems; (c) effectiveness considerations and socio-structural contexts of HIV treatment experiences within broader implementation contexts.

**Conclusion:**

Ongoing research and normative deliberation is required in order to successfully and ethically scale-up TasP within the continuum of HIV care models. Based on the results of this scoping review, we identify several ethical and implementation dimensions that hold promise for informing the process of scaling up TasP and that could benefit from new research.

## Background

The role of antiretroviral therapy (ART) in preventing the onward spread of HIV is now widely accepted as a crucial component within comprehensive HIV prevention efforts [1] based on a variety of empirical findings, including those stemming from mathematical modeling [2], population-level surveillance studies [3,4] and clinical trials [5]. Thus, ‘treatment as prevention’ (TasP) has been positioned as a ‘double-hat trick’ [6] based on ART’s role in preventing disease progression among HIV-positive individuals, as well as population-level benefits of decreasing community-level viral loads and the likelihood of onward transmission to uninfected partners [3,7]. Despite the evidence showing the promise of TasP in reducing HIV incidence, scientists, community activists and government officials have raised ethical questions regarding the implementation and scaling-up of TasP. To date, however, the scope of these discussions in the literature has remained largely unexamined [8], with the exception of a recent systematic review of a particular set of ethical issues associated with clinical and health service challenges related to the HIV “test and treat” strategies [9].

Given the high profile and potential promise of TasP in combatting the global HIV epidemic, an explicit and transparent research priority-setting process is critical to meaningfully inform ongoing ethical discussions pertaining to TasP. This strategy requires a comprehensive and systematic synthesis of the key ethical issues and concerns related to the implementation and scaling up TasP as expressed by both scientists and community stakeholders. For example, within the TasP approach, clinical treatment guidelines within many settings now recommend ART for all individuals living with HIV, regardless of their individual CD4 count or based on an expanded set of parameters^a^. This raises questions about the kinds of efforts that will be required within these large-scale programs to identify and engage HIV positive individuals into long-term care and treatment. As a result, questions related to an individual’s autonomy and the potential for coercive seeking and testing strategies during implementation warrant examination [9]. Moreover, there has been increasing concern that these efforts are being advanced for the benefit of future populations, rather than for the benefit of individuals who are currently HIV seropositive [9]. Despite criticisms that there is insufficient existing scientific evidence to substantiate the trend towards ‘early’ initiation of ART based on individual-level benefits and/or burdens, recent evidence from an ongoing clinical trial (HPTN 052) points to a set of individual-level benefits to early uptake of ART, such as delayed time to onset of AIDS and decreased incidence of primary and secondary infections [10]. Nonetheless, there are increasing calls for more evidence in order to better understand these and other issues pertaining to the ethical implementation and scale up of TasP, as well as the broader social and structural forces that shape individual experiences of TasP [11].

In this paper, we draw on concepts and approaches from the field of population and public health ethics to critically interrogate TasP approaches in order to more fully consider and negotiate a balance of individual and collective interests and health outcomes [12]. Our aim is to provide strategic direction in this field by systematically identifying and highlighting key ethical questions pertaining to implementation and scale-up of TasP that will benefit from new empirical research and theoretical advances.

## Methods

### Search strategy and results

We drew on the Arksey and O’Malley framework for conducting scoping review studies [13,14] as well as systematic approaches to identifying empirical and theoretical gaps within ethical discussions pertaining to population-level intervention implementation and scale up [15]. We searched the health science database PubMed to identify relevant peer-reviewed articles on ethical and implementation issues pertaining to TasP. We included English language articles that were published after 2009 (i.e., after the emergence of causal evidence within this field) by using the following search terms: *HIV*, *HIV infections*, *treatment as prevention, TasP, HIV prevention,* and *ethics*. We included review articles, and used citation tracking to identify other relevant sources. We included only articles that included a discussion of the ethical issues pertaining to the implementation and/or scaling up of TasP. Articles that only discussed issues pertaining to TasP research (e.g., ethical issues that arise in randomized controlled trials) were excluded, as well as articles that focused exclusively on other HIV prophylaxis regimes (e.g., PreP). Empirical studies that did not engage with implementation/scale up or ethical issues were excluded.

Searches yielded 374 results in PubMed. We reviewed all abstracts for content, removed duplicates, and included those articles related to the ethical and implementation issues related to TasP, resulting in a total of 19 articles. We also hand searched the references of relevant articles and special journal issues within this field, and co-authors searched their own library archives, resulting in additional articles, for a total of 8 peer-reviewed articles. Finally, one article was added via citation chasing and one from the library of a reviewer, resulting in a total of 29 articles.

Given the tendency for the exclusion of grey literature within systematic reviews [16], as well as the indication that much of the criticism of TasP occurs outside the peer-reviewed literature (e.g., selection bias), we decided the inclusion of grey literature would provide a more exhaustive representation of how the ethical discussions pertaining to TasP have and are currently taking place. In order to identify relevant grey literature, we used methods outlined by the *The Centre for Reviews and Dissemination*[17]. This included searching various search engines (e.g., google), databases (e.g., CATIE), and webpages (e.g., World Health Organization’s webpage), for the above listed keywords. This search yielded 26 results (12 online articles, 6 reports, 5 position statements, and 3 power point presentations). Including only those sources that explicitly discussed the ethical and implementation issues of TasP resulted in a final list of 11 online articles, 3 reports, 3 position papers, and 2 power point presentations.

### Data extraction and analysis

Co-authors BP and RK screened the search results for eligibility. Data containing ethical discussions of TasP advanced within the peer-reviewed and grey literature were extracted and managed in Microsoft Excel. Tables 1 and 2 list the peer-reviewed articles and the grey literature (respectively) included in our scoping review. During data extraction and analysis, we focused on identifying excerpts from the included documents that pertain to one or more of the following three questions: (1) What are the dominant moral concerns associated with the scaling up and implementation of TasP?; (2) What are the empirical and/or theoretical gaps that are either implicitly or explicitly revealed within these discussions?; and (3) To what extent is evidence characterized or used to morally justify or prohibit various public health practices pertaining to TasP, and where might additional research and evidence yield benefits in moving these discussions towards theoretical or dialogical consensus?

**Table 1:** Peer-reviewed publications included in the scoping study

**Primary authors**	**Ethical issues emphasized**	**Setting**	Method*
Bärnighausen T, Salomon JA, Sangrujee N: **HIV Treatment as Prevention: Issues in Economic Evaluation.***PLoS Med* 2012, **9**:e1001263.	A, B, C	Worldwide	Commentary
Bärnighausen T, Tanser F, Dabis F, Newell M-L: **Interventions to improve the performance of HIV health systems for treatment-as-prevention in sub-Saharan Africa.***Current Opinion in HIV and AIDS* 2012, **7**: 140–150.	C	Sub-Saharan Africa	Rapid Review
Barr D, Amon JJ, Clayton M: **Articulating a rights-based approach to HIV treatment and prevention interventions**. *Current HIV research* 2011, **9**: 396–404.	A, B	Worldwide	Commentary
Chan R: **Biomedical Strategies for Human Immunodeficiency Virus (HIV) Prevention? A New Paradigm.***Ann Acad Med Singapore* 2012, **41**: 595–601.	A, B, C	Worldwide	Review
Chang LW, Serwadda D, Quinn TC, Wawer MJ: **Combination implementation for HIV prevention: moving from clinical trial evidence to population-level effects.***Lancet Infect Dis* 2013, **13**:65–76.	C	Sub-Saharan Africa	Review
Chen Y: **Treatment-Related Optimistic Beliefs and Risk of HIV Transmission: A Review of Recent Findings (2009–2012) in an Era of Treatment as Prevention.***Curr HIV/AIDS Rep* 2012, **10**: 79–88.	C	Worldwide	Review
Cohen T, Corbett EL: **Test and treat in HIV: success could depend on rapid detection.***The Lancet* 2011, **378**: 204–206.	A, B, C	Sub-Saharan Africa	Commentary
Dawson L: The **Devil in the Details: Thorough Assessment of Evidence and Ethics Is Needed in Evaluating New HIV Prevention Methods.***Am J Bioethics* 2012, **12**: 33–34.	A, C	Worldwide	Commentary
Dennin RH, Lafrenz M, Sinn A, Li L: **Dilemma of concepts and strategies for the prevention of spread of HIV in relation to human behavior, law and human rights.***J Zhejiang U* 2011, **12**: 591–610.	A	Worldwide	Commentary
Forsyth AD, Valdiserri RO: **Reaping the prevention benefits of highly active antiretroviral treatment.***Current Opinion in HIV and AIDS* 2012, **7**: 111–116.	A, B, C	Worldwide	Commentary
Garnett GP, Baggaley RF: **Treating our way out of the HIV pandemic: could we, would we, should we?***The Lancet* 2009, **373**: 9–11.	A, C	Worldwide	Commentary
Gupta RK, Wainberg MA, Brun-Vezinet F, Gatell JM, Albert J, Sonnerborg A, Nachega JB: **Oral Antiretroviral Drugs as Public Health Tools for HIV Prevention: Global Implications for Adherence, Drug Resistance, and the Success of HIV Treatment Programs.***J Infect Dis* 2013, **207**: S101–S106.	C	International	Commentary
Haire BG: **Because we can: clashes of perspective over researcher obligation in the failed PrEP trials.***Dev world bioethics 2011,***11**: 63–74.	A, B, C	Worldwide	Commentary
Haire BG, Kaldor J, Jordens CFC: **How good is “good enough”? The case for varying standards of evidence according to need for new interventions in HIV prevention.***Am J Bioethics* 2012, **12**: 21–30.	A, B, C	Worldwide	Commentary
Krellenstein J, Strub, S: **The ethical implications of “treatment as prevention” in the United States.** 2012, **16**:1–4.	A, C	United States	Commentary
Kulkarni SP, Shah KR, Sarma KV, Mahajan AP: **Clinical Uncertainties, Health Service Challenges, and Ethical Complexities of HIV “Test-and-Treat”: A Systematic Review.***Am J Public Health* 2013, **103**: e14–e23.	A, B, C	Worldwide	Systematic Review
Macklin R, Cowan E: **Given financial constraints, it would be unethical to divert antiretroviral drugs from treatment to prevention.***Health Aff* 2012, **31**:1537–1544.	A, B	United States	Commentary
McNairy ML, Cohen M, El-Sadr WM: **Antiretroviral Therapy for Prevention Is a Combination Strategy.***Curr HIV/AIDS Rep* 2013, **10**: 152–8.	B, C	Worldwide	Commentary
McNairy ML, Howard AA, El-Sadr WM: **Antiretroviral Therapy for Prevention of HIV and Tuberculosis: A Promising Intervention but Not a Panacea.***JAIDS* 2013, **63**: S200–7.	B, C	Worldwide	Commentary
Mayer KH: **Antiretrovirals for HIV prevention: translating promise into praxis.***The Lancet* 2011, **378**:206–208.	B, C	Sub-Saharan Africa	Commentary
Montaner JS: **Treatment as prevention: a double hat-trick.***The Lancet* 2011, **378**:208–209.	C	Canada	Commentary
Nguyen V-K, Bajos N, Dubois-Arber F, O’Malley J, Pirkle CM: **Remedicalizing an epidemic: from HIV treatment as prevention to HIV treatment is prevention.***AIDS* 2011, **25**:291–293.	A, C	Worldwide	Commentary
Ostmann F, Saenz C: **Separate Goals, Converging Priorities: On the Ethics of Treatment as Prevention.***Developing World Bioethics* 2013, **13**:57–62.	A, B	Worldwide	Commentary
Singh JA: **Antiretroviral resource allocation for HIV prevention.***AIDS* 2013, 27:863–865.	B, C	Worldwide	Commentary
Small W, Kerr T: **HIV Treatment as Prevention and the Role of Applied Social Science Research.***Journal of AIDS & Clinical Research* 2013, **02**:102e.	B, C	Worldwide	Commentary
Cates W: **HPTN 052 and the future of HIV treatment and prevention.***The Lancet* 2011, **378**: 224–5.	A, B, C	Worldwide	Correspondence
**Lancet Infectious Diseases: Editorial: Treatment as prevention for HIV**. *The Lancet Infect Dis* 2011, **11**:651.	B, C	Worldwide	Commentary
Vonn M: **British Columbia’s ‘seek and treat’ strategy: a cautionary tale on privacy rights and informed consent for HIV testing**. *HIV/AIDS Policy & Law Review* 2012, **16**:1–4.	A, C	Canada	Commentary
Williams B, Wood R, Dukay V, Delva W, Ginsburg D, Hargrove J, Stander M, Sheneberger R, Montaner J, Welte A: **Treatment as prevention: preparing the way**. *JIAS* 2011, **14**:S6.	A, B, C	Worldwide	Commentary

*Only articles that included methods were considered reviews. Articles without methods were considered commentaries.

A = Balancing individual- and population-level interests.

B = Power relations within clinical practice and competing resource demands within health care systems.

C = Effectiveness considerations and socio-structural contexts of HIV-related experience.

**Table 2:** Grey literature reviewed in the scoping study, including the source and title

**Year**	**Title**	**Document type**	**Source organization**	**Ethical issues emphasized**	**Setting**
2013	Mapping pathways: Developing evidence-based, people-centred strategies for the use of antiretrovirals as prevention	Report	Rand Website	C	Worldwide
2012	Treatment as Prevention: recognising the creative potential of antiretroviral medications	Online article	HIV, Science, and the Social	A, B	Worldwide
2013	Reactions to the test-and-treat models	Online article	NAM AIDS MAP	A, C	Worldwide
2012	Emerging Issues in Today’s HIV Response: Debate 6 Treatment as Prevention	Report	AIDSTAR-One	B, C	Worldwide
2013	The Human Rights Issue	Online article	NAM AIDS MAP	A, B	Worldwide
2010	Treatment as Prevention: Protecting Individual Autonomy	Online article	aidsperspective.net	A, B	Worldwide
2009	Ethics of ART for HIV Prevention as a Public Health Intervention	Presentation	WHO	A, B, C	Worldwide
2009	Consultation on Antiretroviral Treatment for Prevention of HIV Transmission: Meeting Report	Report	WHO	B, C	Worldwide
2013	Changing my mind on treatment as prevention	Online article	Positive Lite	A, C	Worldwide
2010	Prominent Parisian Activist Weighs in on Treatment as Prevention	Online article	POZ Blogs	A	Worldwide
2010	Views from the front lines: Treatment as Prevention	Online article	CATIE	B, C	Canada
2013	Treatment as prevention: Bob Leahy in conversation with James Wilton	Online article	CATIE	A, C	Canada
2012	Treatment benefits for all?	Online article	CATIE	A, B	Canada
2009	Antriretroviral treatment for prevention	Position statement	UN AIDS	A	Worldwide
2012	Controlling the HIV Pandemic with Antiretrovirals: Treatment as Prevention and Pre-Exposure Prophylaxis - Consensus Statement	Position statement	IAPAC	A, B, C	Worldwide
2013	The use of antiretroviral therapy to reduce HIV transmission	Position statement	BHIVA, EAGA	C	Worldwide
2010	Biomedical Prevention Is Always About Social Justice, Too	Online article	HIV Research Catalyst Forum	A	Worldwide
2010	USPHS guidelines: We need reliable evidence to justify an earlier start of anti-retroviral therapy	Online article	aidsperspective.net	C	Worldwide
2011	Are people living with HIV less likely to pass HIV to others if they are on treatment? Exploring the use of treatment as prevention	Presentation	CATIE	A	Worldwide

A = Balancing individual- and population-level interests.

B = Power relations within clinical practice and competing resource demands within health care systems.

C = Effectiveness considerations and socio-structural contexts of HIV-related experience.

Using these questions as a rubric, we were guided by both deductive and inductive approaches as we employed techniques from thematic analysis to identify patterns and themes within the literature [18]. The co-authors adopted an iterative process throughout the analysis, so that early data analysis informed our subsequent decisions about interpreting the materials. Together, we developed interpretations about recurring, converging and contradictory themes within the data. As the analysis progressed, we compared and contrasted the emergent themes and identified the various rhetorical techniques that were employed. Key concepts within each theme, along with examples from the literature, are presented below.

## Results

**Figure 1:**
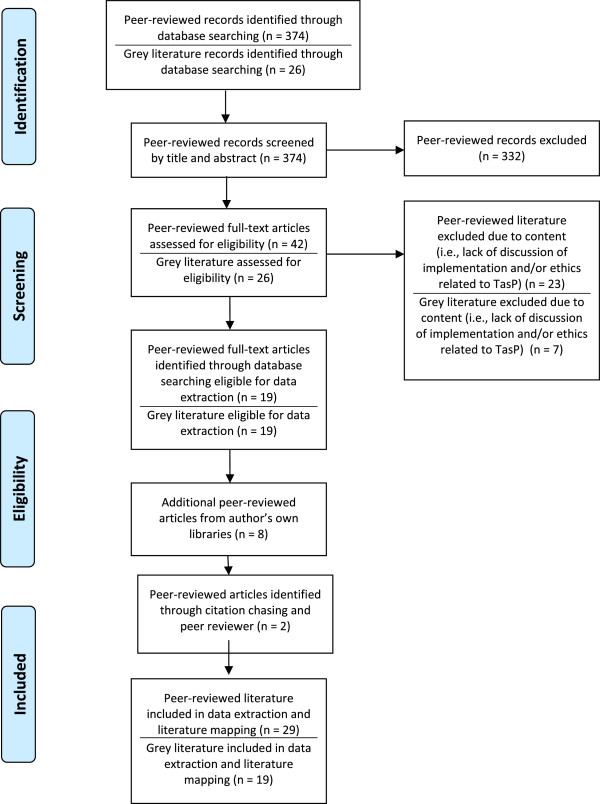
Flow-chart of articles included and excluded.

Our search resulted in 29 peer-reviewed articles and 19 grey literature sources that were included in data extraction and literature mapping. Figure 1 presents a modified version of the PRISMA-style flow diagram to reflect our inclusions/exclusion process^b^. Three dominant themes emerged in our analysis with respect to the ethical questions pertaining to TasP implementation and scale-up: (a) balancing individual- and population-level interests; (b) power relations within clinical practice and competing resource demands within health care systems; (c) effec tiveness considerations and socio-structural contexts of HIV-related experiences within broader implementation contexts.

### Balancing individual- and population-level interests

The ethical implications of initiating individuals on ART regardless of their CD4 count emerged as the most salient and contentious question related to the ethical implementation of TasP [9]. This debate centers on whether or not it is ethically justifiable to initiate an intervention (ART) focused on an individual to derive public health benefits, rather than for the benefit of the individual. Individualistic frameworks, communitarian and utilitarian frameworks are used to advance arguments in this debate.

Individualistic (e.g., social libertarian) frameworks tend to prioritize individual autonomy against a set of communitarian frameworks that emphasize the interests of the broader population [19]. Applications of individualistic frameworks suggest that scaling up TasP programs will result in overly coercive testing and treatment regimes that disregard voluntary consent and participation. Within these arguments, it is suggested that TasP represents an intervention on individuals that burdens individuals in order to promote public health [9], thereby violating principles of beneficence, non-maleficence and autonomy – hallmarks of applied medical ethics [20]. For example, the early initiation of ART is largely positioned as a negative option for the individual due to the potential for various side effects, including altered lipids, reduced bone density and kidney damage [21] as well as the potential for increased antiretroviral resistance [21]. Thus, these arguments suggest that an individual’s choice not to commence treatment confers an individual benefit that outweighs concerns for public beneficence [21]. Additional arguments that suggest individual autonomy trumps overall beneficence rest on the notion that the long-terms effects of ART adherence are not yet fully known. For example, some have suggested that TasP burdens individuals with chronic disease management at an early stage of their disease progression, thereby altering their capacity for economic productivity as well as their overall quality of life [22].

Questions about the ethics of early initiation of ART have also been closely embedded in matters of agency. If the individual freely chooses to initiate TasP (e.g., they have a sufficient level of autonomy, the process remains voluntary, and they are equipped with sufficient information to make an informed choice), then TasP meets the ethical criteria set out within applied medical ethics – regardless of the potential for individual or population-level harms/benefits [8,21]. However, the positioning of agentic choice as devoid of contextual and structural factors has also been problematized. These arguments reveal the dependence of truly free choice on a variety of conditions, including whether or not a person will have the capacity to adhere, whether or not there will be a consistent and ‘never-ending’ supply of ART available [23], and if an individual will have the opportunity to start ART at a later date if they choose to decline early initiation [19]. Finally, the potential for the stigmatization of individuals or groups who choose *not* to seek early treatment was also described as an important ethical consideration regarding the successful scaling up of TasP [24,25].

Communitarian and utilitarian framings, on the other hand, offer a view that more strongly features the potential for TasP to prevent future cases as a primary consideration, counter-positioning previous HIV prevention approaches as ‘status quo’ and insufficiently equipped to decrease the spread of HIV [26]. These ‘greater good’ perspectives are often buttressed in the literature by arguments that turn on the extent to which the individual who uptakes ART could benefit (or at least not suffer) as the short-term effects of the early initiation of ART have not been demonstrated to be physically detrimental [27]. The view that populations and individuals may concomitantly benefit from beginning ART with CD4 counts above the previously defined thresholds for treatment has also been extended to the psychological realm (e.g., feeling relief in knowing one is less infectious and therefore less likely to transmit HIV to partners) [21] as well as other potential physiological benefits [27]. While detailed discussions regarding some of the mainstays of population and public health ethics (e.g., relational frameworks that emphasize notions of ‘the greater good’) did not feature as strongly in the literature captured in the current review, some authors did apply key principles, such as solidarity, stewardship and compassion, and argue that these principles ought to be considered in terms of their relevance to population and public health ethics – particularly with regard to concerns pertaining to the most vulnerable population sub-groups [28]. Some arguments appear to minimize the potential risks for individuals in the face of potentially large population-level gains. For example, it has been suggested that the addition of two to three years of treatment (e.g., before the immune system becomes compromised) could be considered as insignificant in the face of what could be decades of future treatment [27].

### Power relations within clinical practice and competing resource demands within health care systems

The second dominant theme that emerged in the analysis related to power relations within patient-physician interactions and health care systems more broadly [9]. For example, some authors have suggested that without unequivocal evidence pertaining to the early uptake of ART, doctors will still be faced with the challenge of providing advice to their patients on courses of action in a context of uncertainty [29]. Particularly in situations where the evidence is either contradictory or unavailable (e.g., issues pertaining to the individual-level benefits of early uptake of ART), the ethical and effective negotiation of power relations inherent in patient-physician interactions will require a relatively high degree of sophistication from both parties [25,30]. Thus, the importance of training of health care practitioners to competently relay evidence about the potential risks of early uptake of ART was also identified as an important component to ensure individuals can make informed decisions. However, inherent difficulties within the doctor-patient relationship regarding comprehension and translation of risk information were acknowledged (particularly in light of limited or conflicting evidence in this area) [31].

Questions also have been raised in the literature around the prioritization and distribution of limited resources. Indeed, in the context of scarce resources, issues related to TasP have led some to question the extent to which efforts should be focused on treatment *versus* prevention [32]. For example, some authors have suggested that, given a limited set of resources in HIV treatment and prevention programs, it is inequitable to divert resources from treatment to prevention [33] – particularly in relation to taking ARV treatment resources away from resource-poor settings in order to advance prevention efforts within high-resource or low-prevalence settings [34,35]. Still others argued that the very nature of TasP should be seen as paradigm-shifting and that the goals of treatment and prevention are converging rather than conflicting [32]. Moreover, the unknown issues pertaining to the long-term preventative effects of TasP have been said to be so significant that predicting the associated costs and benefits is not yet possible [22]. Ultimately, the treatment versus prevention debates tend to flow from utilitarian frameworks which argue for the prevention of the greatest number of deaths versus deontological frameworks which argue for ethical rules (e.g., the rule/duty of rescue) and prioritarian principles (e.g., arguing that those who are currently sick are worse off than those who will become sick but are currently healthy) [28,33].

Issues related to the scarcity of HIV prevention and health promotion resources have also led to a set of arguments around targeting efforts at specific population sub-groups considered most at-risk for transmitting HIV for cost-specific reasons [36], though many acknowledge the ethical complexities associated with deciding how efforts ought to be prioritized during implementation [37,38]. For example, the literature generally acknowledges that population sub-groups that already face the highest levels of stigmatization (e.g., people who inject drugs) are likely the most at risk for HIV acquisition and transmission [39]. This presents a challenge regarding the population-level effectiveness of TasP, as stigma also deters people from accessing regular testing, thereby delaying the onset of diagnosis and treatment, and undermining the potential for reduced transmission [40]. As a result, some have called for more research on the efficacy of TasP in marginalized sub-groups in order to inform ways to ethically reach people at the earliest stages of infection (i.e., during the first 6 months) when their viral loads may be highest [36,40].

Others have suggested that explicit criteria be used in order to determine who should be prioritized to receive treatment. In general, children and monogamous sex partners (those least likely to transmit the virus) are characterized as low priority groups for TasP, whereas those who are the most likely to spread HIV should be high priority (e.g., men who have sex with men in high-risk settings; people who inject drugs; migrant workers with multiple concurrent partners) [39]. These arguments are premised on the assumption that treatment is a finite resource and offering treatment to somebody who is not sick (e.g., individuals with a CD4 count well above 350 and not showing symptoms of HIV infection) may deprive a sick person (e.g., an individual with a CD4 count well below 200 and experiencing HIV-related symptoms) of treatment. As a result, the principle of providing treatment to those individuals who will benefit the most comes into conflict with principles related to health maximization in which the HIV negative partner in a sero-discordant relationship will benefit [34].

TasP is most widely referred to as one strategy within a set of HIV prevention programs, often referred to as a continuum, cascade or combination prevention strategy [1,33,36,40,41]. In addition to TasP, other HIV intervention strategies within health care systems include voluntary medical male circumcision, campaigns to increase HIV testing rates, condom availability and (in some settings) pre- and post-exposure prophylaxis (i.e., PEP and PreP) [38,40-42]. As a result, the successful and ethical implementation of TasP relies heavily on the capacity of health systems to accommodate its introduction. Key questions identified here concern issues pertaining to testing, effective linkage to care for those who test positive, retention in care, initiation of ART and assistance with adherence issues to attain viral suppression [41]. Given that the successful implementation of TasP requires a large increase in the uptake of HIV testing, prevention, treatment and support services, questions have been raised regarding the capacity of some health systems (which are ultimately embedded within larger socio-political systems) to introduce TasP in ways that ensure human rights are protected, HIV-related stigma and discrimination are reduced (or at least not exacerbated), and to facilitate a sustained and ethical engagement of the individuals accessing TasP-related programs over very long periods of time (e.g., indefinitely) [8,24].

### Effectiveness considerations and socio-structural contexts of HIV-related experiences

The third emergent theme centered on questions related to the external validity of randomized controlled trials supporting the role of TasP, leading to concerns about whether existing trial data is sufficient to justify the widespread implementation of TasP [20,37,43]. While some argue that the findings of clinical trials support offering ART to individuals in sero-discordant relationships [6], other evidence has suggested that a large proportion of new HIV infections do not occur within stable sero-discordant heterosexual relationships [38]. Thus, questions have emerged as to whether or not the evidence base warrants the implementation and scale up of TasP within epidemics that are not defined by vaginal-penile transmission [36]. Others have suggested that while the efficacy of TasP in clinical trials settings is acceptable, effectiveness considerations may render the approach’s ‘real-world’ effectiveness vulnerable to criticisms (e.g., effect sizes will diminish to non-clinically significant levels in the face of broader implementation realities) [24,36]. Consequently, some have argued that the ethical evaluation of evidence to policy requires more than data from clinical trials and that a justification to proceeding to widespread scale up must also be informed by information pertaining to the social and economic realities of the implementation contexts [37], including community-based effectiveness trials that may better account for the heterogeneity of experiences for people living with HIV [23].

It also has been postulated that effectiveness studies could reveal TasP as having unintentional inequity-enhancing consequences. For example, effectiveness trials might demonstrate the potential for a skewing of beneficial impacts among various population sub-groups (e.g., differential uptake across social strata; variegations in the number and strength of barriers to access along the social gradient) [22,44]. Unintended effects on individual-level behavior were also discussed in the literature, particularly risk compensation behavior (e.g., “condom migration” effects) [24,42,45,46]. The potential for risk compensation has led some to ask how this effect could potentially undermine or blunt the preventative aspects of TasP, reinforcing arguments that point to the necessity of maintaining and perhaps enhancing current standards of HIV prevention (e.g., risk-reduction counseling in clinical settings) in TasP implementation and scale-up [46].

The issue of adherence in ‘real world’ settings was widely discussed in the literature, with many suggesting that the levels of adherence attained in clinical trials cannot be attained within most settings globally – particularly within resource-poor settings that present a set of geographical and sociocultural challenges [24,44,47,48]. For example, some have argued that evidence pertaining to adherence must be contextualized to local settings [37,41,49], with particular attention focused on the social vulnerabilities that individuals may experience in their everyday lives (e.g., unstable housing) [8,24,31,33,44,47,48] . As such, specific implementation challenges were identified regarding engaging around TasP with individuals who already face social, cultural and economic barriers to accessing care [49]. For example, one article described possible scenarios in which some vulnerable individuals (e.g., people who inject drugs) may become “ghettoized” in that they can only live in places that support adherence to treatment [50]. And, it has been suggested that if the scale up and implementation of TasP is only partially successful, it could potentially concentrate future infections within highly vulnerable populations, thereby raising serious ethical concerns related to health equity and social justice [19]. Questions also have been raised regarding the ethical implications of incentivizing HIV testing (e.g., monetary compensation), particularly within seek and treat models targeted to vulnerable population sub-groups [51]. Finally, some have suggested that TasP approaches that induce treatment initiation ‘early’ may unintentionally incur system-wide opportunities for patient non-compliance with drug regimens, which therefore heightens the risk of additional drug resistance and limits future drug treatment options [21].

Given these concerns, the social determinants of health were often cited as a key priority within the literature. For example, organizations such as UNAIDS and the World Health Organization recommend comprehensive approaches to HIV treatment and prevention that also acknowledge and respond to the underlying and upstream causes of HIV infection, including gender inequality and access to health care, employment and housing [49,52]. In some instances the social determinants of health were positioned against the remedicalization of the HIV epidemic through the use of biomedical interventions such as TasP; these arguments suggest that, without paying sufficient attention to the underlying causes of vulnerability and HIV infection, TasP will have only limited success [51]. Conversely, TasP is positioned as having already been grounded in the social and structural dimensions of HIV-related experiences. For example, in some implementation settings, legal and policy barriers (e.g., the criminalization of HIV; absence of evidence-based HIV prevention and addiction treatment opportunities or programs) are positioned as key ‘forces’ that will contribute to the differential uptake/success of TasP, thereby exacerbating health inequity – particularly among already vulnerable population sub-groups (e.g., people who inject drugs) [11].

Some of the most impassioned arguments in the literature related to the socio-structural contexts of HIV-related experiences, with some authors characterizing TasP’s commitments to addressing the distal causes of HIV as rhetoric [8]. For these authors, TasP’s capacity to be effective requires ongoing commitments to human rights, including the provision of access to non-discriminatory services, policies and laws [8]. In particular, laws that criminalize HIV transmission have been identified as a significant socio-structural barrier to the ethical and effective scaling up of TasP, as well as other HIV-related interventions in which TasP is implicated (e.g., routine HIV testing) [8,42].

## Discussion

In the current scoping review, a variety of normative ethical theories were either implicitly or explicitly used to ground the arguments advanced in the literature regarding the implementation of TasP. Specifically, we explored three key themes that have important ethical and implementation implications: (1) Effectiveness considerations and socio-structural contexts of HIV-related experiences; (2) Power relations within clinical practice and competing resource demands within health care systems; and (3) Balancing individual- and population-level interests. While conflicting interests associated with various ethical frameworks may not be fully resolvable (e.g., utilitarian versus deontological approaches), ongoing discussion and deliberation in this area will help provide progress towards a reflective equilibrium in which a degree of ethical coherence and acceptability is acquired. Below we outline where priorities for empirical research will advance these ethical discussions. Based on the results of this scoping review, we identify several areas that hold promise for informing the ethical implementation and scale up of TasP and that could benefit from new research (see Table 3 for an abbreviated list of key recommendations from these findings).

**Table 3:** Priorities for future research

1	**Effectiveness studies that integrate ethical dimensions of TasP implementation:** Examining the social, economic and political contexts that influence the capacity for successfully and ethically implementing and scaling up TasP.
2	**Examinations of power relations in clinical interactions:** Empirical research examining the conditions that enable patients to make fully informed decisions about TasP that are free of coercion.
3	**TasP as a case study of ‘targeting’:** Ongoing empirical and philosophical work should aim to characterize the extent to which TasP differentially affects population sub-groups.
4	**Empirical estimations to inform resource distribution decision-making in the context of natural experiments:** Move beyond the ongoing ‘treatment versus prevention’ debate and focus energies on developing new empirical estimations regarding the costs (including opportunity costs) of scaling up TasP.
5	**Public engagement and interdisciplinary science:** Use techniques associated with implementation and social science (e.g., fieldwork; interviews; policy analysis) and moral frameworks from the field of population and public health ethics (e.g., social justice frameworks).

### Effectiveness studies that integrate ethical dimensions of TasP implementation

Many questions about the ethical implementation of TasP are based on a set of uncertainties surrounding its scale up beyond efficacy trials. These questions tend to focus on the potential for unintended effects of TasP on individuals (e.g., long-term effects on those initiating early ART) as well as on population subgroups (e.g., further stigmatization of people who inject drugs) [11]. Ongoing experimental research such as the START (Strategic Timing of Antiretroviral Treatment; clinical trial NCT00867048) will provide important information related to the individual-level physiological effects of early ART initiation (e.g., long-term side effects) though these findings will not be available until at least 2015. Findings from clinical trials represent an important first step in developing new and much-needed evidence. Embedding various research approaches within clinical trials (e.g., using a variety of social science research methods, including qualitative and ethno-epidemiological approaches) will bridge some of the knowledge gaps identified in our review by studying the social, economic and political contexts that influence the capacity for successfully and ethically implementing and scaling up TasP.

There also is an urgent need to support new empirical research that characterizes how interactions between TasP and its various implementation contexts affect the health and social well being of individuals as well as populations. Limited data exist on issues pertaining to risk compensation behavior and the capacity for non-adherence or early initiation to lead to drug resistance (despite many of the ethical arguments being premised on this concern) [51]. While we acknowledge the challenges related to studying risk compensation (e.g., experimental designs in this area are themselves unethical to undertake) [53,54], research efforts focusing on the relationship between treatment, adherence and HIV risk behavior is urgently required in order to advance more fully informed discussion in this area. Nonetheless, emerging evidence in this area is showing a strong connection between social and structural influences on adherence among vulnerable populations, including the legal context of HIV (e.g., HIV non-disclosure legislation) [55]. Indeed, within the current scoping review, social vulnerability and economic disadvantage emerged as a dominant ethical concern. As such, issues pertaining to vulnerability require careful and context-specific empirical and normative analyses that can extend to many of the upstream issues often considered outside of the realm of intervention research methodology (e.g., randomized controlled trials; pharmaceutical research). Adopting techniques from the realm of science and technology studies (STS) may help reveal how implementation and ethical considerations are inextricably linked and embedded in one another. For example, the field of STS emphasizes the connections between knowledge about human biology and technologies (e.g., TasP) alongside the ‘social’ (e.g., social contexts) and the ‘ethical’ (e.g., normative theory).

In addition, effectiveness studies offer opportunities to embrace heterogeneous experiences associated with TasP implementation as well as the social and structural factors that shape HIV-related experiences – particularly among vulnerable and socially disadvantaged population sub-groups. While clinical trials have provided information related to heterosexual sero-discordant couples, additional information related to men who have sex with men and other vulnerable population sub-groups (e.g., people who inject drugs) is urgently required in order to advance these discussions. Highly-controlled clinical trial settings with primarily homogenous samples do not have sufficient capacity to detect the diversity of pathways and mechanisms through which TasP might be implemented across contexts; thus, relying exclusively on information regarding implementation and ethical considerations gleaned from controlled trials may point us in unrealistically uniform future directions. Furthermore, homogenous samples effectively limit (by default) forms of variability that are important considerations in ethical implementation, including behavioural characteristics, cultural and ethnic traditions and gender norms. Building capacity in effectiveness studies also could enhance the likelihood of undertaking international comparative studies to further determine the extent to which context-based heterogeneity (e.g., different forms of health systems) shape or respond to the ethical and implementation dimensions of TasP.

### Examinations of agency, including agentic practices that may challenge dominant social norms

Many authors argued that if an individual is fully informed and has the capacity to consent (e.g., is theoretically ‘autonomous’ within clinical interactions) to the early initiation of TasP, public health practices associated with TasP are ethically justifiable. However, many questions arose as to whether all individuals exposed to TasP will have the capacity to be fully autonomous given the power relations manifest in many clinical interactions – particularly among vulnerable or disadvantaged population sub-groups. In addition, while the literature reviewed here does not surface concerns that clinicians might make a set of idiosyncratic ‘choices’ (e.g., those influenced by prejudices) about who should or should not be offered TasP, these concerns have been identified in another body of literature [56] – that pertaining to the allocation of ART. The roles that prejudice or discrimination play in relation to the scaling up of TasP as yet remain unclear and merit further study. Empirical research in this area is needed in order to fully distil the conditions that provide patients with fully informed decisions that are free as possible from coercion. For example, drawing on methods and theories from the field of empirical bioethics, empirical data can be generated to explore the extent to which clinical interactions pertaining to TasP are voluntary [57]. Based on new evidence, there may be opportunities to either improve the ethical theory or change the clinical practices in order to attain the demands of specified ethical principles, particularly when agentic practices of individuals or groups run counter to dominant social norms [58].

### TasP as a case study of ‘targeting’

Within the realm of public health, there is concern that features of various targeted public health interventions (whereby a particular subgroup of population is targeted for an intervention or enhanced intervention focus) may exacerbate health inequity [59,60], raising moral concerns regarding their use [61,62]. Targeted interventions have been critiqued for burdening specific population subgroups with an intervention, while aiming to benefit those not explicitly targeted through a *dispersion of benefits*. TasP represents an important area in which more research, evidence and philosophical deliberation may provide some resolution pertaining to the ethical implications of targeted public health practices. In order to provide moral guidance in this area, ongoing empirical and philosophical work should aim to characterize the extent to which a burden negatively impacts individuals and population sub-groups targeted for TasP.

### Empirical estimations to inform resource distribution decision in the context of evolving natural experiments

Perhaps one of the most salient and complex moral dilemmas that arises relates to issues around resource distribution within the context of scarcity and various global austerity measures. Based on our analysis of the literature included in the current scoping review, we suggest it would be productive to move beyond the ongoing ‘treatment versus prevention’ debate and focus energies on developing new empirical estimations regarding the costs (including opportunity costs) of scaling up TasP. To make informed decisions globally and locally about ethical scaling up of TasP, it will be important to understand the degree to which investments in TasP efforts may imply opportunity costs (or have a multiplier effect) within a broader comprehensive spectrum of care and more upstream prevention.

### Public engagement and interdisciplinary science

Within these efforts, interdisciplinary and collaborative approaches are required to engage in normative assessments of various sets of evidence. Indeed, based on these finding, techniques associated with implementation and social science (e.g., fieldwork; interviews; policy analysis) and moral frameworks from the field of population and public health ethics (e.g., social justice frameworks) will play an important role in leveraging the successful and ethical implementation of TasP. Finally, in order to mitigate some of the conflicting arguments in this area, we suggest the source origins of both the methodological and philosophical frameworks should be addressed to the fullest extent possible, along with clear, transparent rationale with respect to how the empirical and normative interacted to arrive at a moral conclusion. Indeed, the evaluation of data in this area should continue to engage in critical approaches, given the inherent difficulty of transforming complex research findings (e.g., population-level studies) into moral conclusions [63].

### Strengths and limitations

This scoping study was limited in its capacity to reveal all ethical issues that will arise within this area (e.g., there could be other publications that were not identified using our search criteria; additional ethical debates pertaining to TasP take place ‘outside’ of the peer-reviewed and grey literature). In addition, articles in languages other than English were not included. Moreover, this study did not appraise the quality of ethical arguments (e.g., based on a logic framework) nor the quality of the evidence base on which various arguments rest. However, by drawing on both peer-reviewed and grey literature, our review does provide illustrations of some dominant ethical issues that demand new empirical work that could inform the ethical implementation and scaling up of TasP.

## Conclusions

New research and normative deliberation is required in order to successfully and ethically treat and prevent HIV through the inclusion of TasP within HIV continuum of care models. From an ethical and implementation perspective, the process of scaling up TasP should not be conceptualized or practiced as linear, but rather as a constant feedback loop in which new evidence constantly informs morally robust practices within and during its implementation.

## Endnotes

^a^Before the use of ART for TasP, the CD4 count (a measure of white blood cells in units of cells/mm^3^) at which individuals were recommended to begin ART was generally accepted to be at approximately 350 cells/mm^3^ or less [64]. Since the initiation of TasP, however, some jurisdictions such as British Columbia, Canada, no longer consider CD4 level thresholds [5,65] with respect to ART initiation. Currently, the World Health Organization recommends a CD4 < 500 cells/mm^3^[66] and some settings (e.g., Australia) do not provide funding for treatment or recommend ART if CD4 count is above specifically defined thresholds (e.g., >500 cells/mm^3^). In other settings (e.g., low-resource settings), ART may be altogether unavailable for prevention purposes [67-69].

^b^While there are similarities between the methods associated with systematic reviews and the methods and techniques of scoping reviews with respect to ‘mapping’ the extent, range and nature of various substantive areas (e.g., TasP), there are also important differences (see: Arksey & O’Malley, 2005; Levac & O’Brien, 2010). As a result, we have modified the PRISMA flow chart to reflect our inclusion/exclusion processes.

## Competing interests

The authors declare that they have no competing interests.

## Authors’ contributions

RK and WS developed the study design and protocol. BP carried out the literature search. BP and RK screened the literature collected for inclusion/exclusion criteria. KT provided analytic contributions to the (re) analysis of data and revisions to the manuscript upon important feedback from reviewers. All authors contributed to the thematic analysis and drafting of the findings. All authors reviewed and approved the final manuscript.

## Pre-publication history

The pre-publication history for this paper can be accessed here:

http://www.biomedcentral.com/1472-6939/15/54/prepub
